# Species Differences in Metabolism of Soluble Epoxide Hydrolase Inhibitor, EC1728, Highlight the Importance of Clinically Relevant Screening Mechanisms in Drug Development

**DOI:** 10.3390/molecules26165034

**Published:** 2021-08-19

**Authors:** Cindy B. McReynolds, Jun Yang, Alonso Guedes, Christophe Morisseau, Roberto Garcia, Heather Knych, Caitlin Tearney, Briana Hamamoto, Sung Hee Hwang, Karen Wagner, Bruce D. Hammock

**Affiliations:** 1 UC Davis Comprehensive Cancer Center, Department of Entomology and Nematology, University of California, Davis, Davis, CA 95616, USA; cbmcreynolds@ucdavis.edu (C.B.M.); junyang@ucdavis.edu (J.Y.); chmorisseau@ucdavis.edu (C.M.); shhwang@ucdavis.edu (S.H.H.); kmwagner@ucdavis.edu (K.W.); 2 EicOsis, 1930 5th Street, Suite A, Davis, CA 95616, USA; 3 Department of Veterinary Clinical Sciences, College of Veterinary Medicine, University of Minnesota, St. Paul, MN 55108, USA; guede003@umn.edu (A.G.); cctearney@gmail.com (C.T.); 4 Dechra Development LLC, 1 Monument Sq, Portland, ME 04101, USA; Roberto.Garcia@dechra.com; 5 K.L. Maddy Equine Analytical Pharmacology Laboratory, School of Veterinary Medicine, University of California, Davis, Davis, CA 95616, USA; hkknych@ucdavis.edu (H.K.); bdhamamoto@ucdavis.edu (B.H.); 6 Department of Veterinary Molecular Biosciences, School of Veterinary Medicine, University of California, Davis, Davis, CA 95616, USA

**Keywords:** soluble epoxide hydrolase, companion animals, pharmacokinetics, feline drug metabolism

## Abstract

There are few novel therapeutic options available for companion animals, and medications rely heavily on repurposed drugs developed for other species. Considering the diversity of species and breeds in companion animal medicine, comprehensive PK exposures in the companion animal patient is often lacking. The purpose of this paper was to assess the pharmacokinetics after oral and intravenous dosing in domesticated animal species (dogs, cats, and horses) of a novel soluble epoxide hydrolase inhibitor, EC1728, being developed for the treatment of pain in animals. Results: Intravenous and oral administration revealed that bioavailability was similar for dogs, and horses (42 and 50% F) but lower in mice and cats (34 and 8%, respectively). Additionally, clearance was similar between cats and mice, but >2× faster in cats vs. dogs and horses. Efficacy with EC1728 has been demonstrated in mice, dogs, and horses, and despite the rapid clearance of EC1728 in cats, analgesic efficacy was demonstrated in an acute pain model after intravenous but not oral dosing. Conclusion: These results demonstrate that exposures across species can vary, and investigation of therapeutic exposures in target species is needed to provide adequate care that addresses efficacy and avoids toxicity.

## 1. Introduction

Companion animal medications are often repurposed drugs approved for human use or for use in species other than the patient being treated [1]. Although more research is being conducted to understand exposures in companion animals, information is still lacking, and limited understanding of the distribution and pharmacokinetic profiles of compounds in the intended species can result in failed efficacy from conservative dosing strategies used to avoid toxicities. Due to the paucity of data, dose recommendations are often based on allometric scaling and in-vitro metabolic stability in microsomes; however, this may not capture true metabolism if other mechanisms of elimination, such as intestinal transporters or phase II metabolism, are involved [2]. The broad generalizations of exposure based on human data or data in other species are especially problematic for cats. Cats are obligate carnivores and have fewer mechanisms for xenobiotic metabolism as a result [3,4]. Court (2013) [5] compared the elimination rate of 25 therapeutics among humans, cats and dogs and found that human elimination rates poorly predicted metabolism in cats and dogs; however, cats and dogs had similar elimination profiles when compounds were excreted unchanged vs. compounds metabolized by CYP450 oxidation or phase II conjugation mechanisms.

This lack of understanding of drug exposures in companion animals often leads to limited treatment options for fear of overdosing, particularly where side effects are a significant concern. This is especially true for pain relief in companion animals [6,7]. Options for pain control in companion animals are limited to a few options, such as non-steroidal anti-inflammatory drugs (NSAIDs), opioids or repurposed seizure medications. While opioids are effective analgesics in companion animals, their use is accompanied by the same severe side effects seen in humans, such as gastric stasis [8]; however, it is commonly assumed that cats and horses respond to opioids with increased activity, termed opioid mania, but this is only present at higher doses [9,10]. In addition, tolerance and hyperalgesia after chronic dosing further limit their long-term use [11]. NSAIDs are often used as pain relieving options in companion animals despite being one of the 10 most common causes of unintentional overdose in dogs and cats [12]. Dogs and horses tend to tolerate NSAIDs at higher doses than cats due to limited glucuronyl transferase in cats, but even dogs and horses are not exempt of potential adverse events when using long-term NSAIDs due to gastro-intestinal, kidney and liver toxicities [13,14]. Recently anti-seizure gabapentinoids, such as gabapentin, are being used to treat pain and seizures in companion animals, and despite being a commonly prescribed pain medication for chronic musculoskeletal pain in cats [15] and highly used in other companion animal species including horses [16], gabapentin has not been approved for use in companion animals, and assessments are on-going to determine safety and efficacy. Sedation and somnolence are the most frequently described dose-limiting side effects, and withdrawal symptoms, such as rebound pain and agitation which are observed in humans, could further complicate their use in companion animals [17,18,19].

Due to limited safe and effective long-term pain-relieving options in companion animals, improved analgesic drugs with a novel mechanism of action are greatly needed. EC1728 is currently under development for treating pain in companion animals based on inhibition of the soluble epoxide hydrolase (sEH) to increase beneficial and natural analgesic epoxy fatty acids (EpFA). Essential polyunsaturated fatty acids (PUFAs) are metabolized primarily through three enzymatic mechanisms, cyclooxygenase (COX), lipoxygenase (LOX) and cytochrome P450 (CYP450) enzymes, that results in the formation of both inflammatory and inflammation-resolving regulatory lipid metabolites. While the COX and LOX pathways form largely inflammatory products, the EpFA formed from CYP450 have been shown to reduce pain and inflammation (for review see [20]). However, the biological relevance of EpFA is limited by their rapid degradation by the sEH into corresponding vicinal diols that are inactive or pro-inflammatory [21]. In contrast to NSAIDs that inhibit the COX enzymes to prevent the formation of inflammatory prostaglandins, inhibition of sEH is being developed as an analgesic option by largely preventing metabolism of beneficial EpFAs. By effecting a response through increasing concentrations of safe endogenous compounds, the on-target toxicity of sEH inhibitors is expected to be less than those of other drug targets. There are many efforts to identify a therapeutic strategy targeting this pathway (for review see [22]), and the primary inhibitors and their physiochemical properties are identified in Table 1. Interestingly, compounds containing a piperidine moiety are significantly less active on the sEH enzyme of dogs and cats. In contract, EC1728 is non-piperidine derivative and selective sEH inhibitor nominated for use in dogs, cats and horses based on its potency, solubility, and stability in vitro [23]. It has shown efficacy in treating both natural disease in horses [24] and dogs [25], and murine models of neuropathic and inflammatory pain [26,27]. EC1728 is a potent inhibitor of the sEH [28], and stable in microsomes, with 100% remaining after 30 min, indicating that the compound is not substantially metabolized by CYP450 enzymes [23]. EC1728 has been evaluated for efficacy and PK in several preclinical species [25,29,30,31]; however, comprehensive PK in target companion animal species has not been specifically characterized. The purpose of this study was to determine exposure of EC1728 after intravenous (IV) and oral dosing in target companion animal species (dogs, cats and horses) to evaluate comparative pharmacokinetics and exposures based on existing literature and new data presented in this paper. Plasma protein binding and in-vitro phase II metabolism studies were conducted to further characterize elimination pathways of EC1728.

## 2. Results

### 2.1. Pharmacokinetic Profiles in Companion Animals

**Figure 1:**
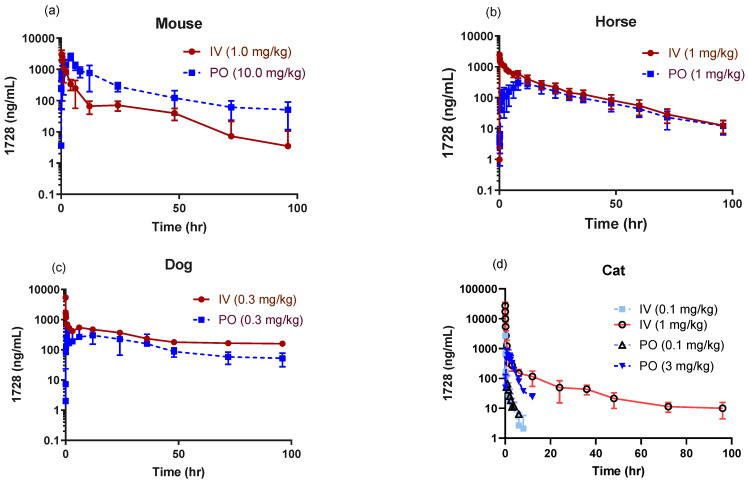
General overview of PK profiles of EC1728 represented as semi log-linear plots of concentrations (mean ± STDEV) after dosing IV or PO in mice (*n* = 4) (**a**), horses (*n* = 8) (**b**), dogs (*n* = 5) (**c**) and cats (*n* = 3−6) (**d**). T_last_ in cats at 0.1 mg/kg IV and PO was 8 h for IV and 12 h for PO; however, the C_last_ in the low dose cat group dosed IV was observed at 3 h and 4 h for PO. Linear representation of graphs through 12 h are plotted in Supplementary Figure S1.

**Figure 2:**
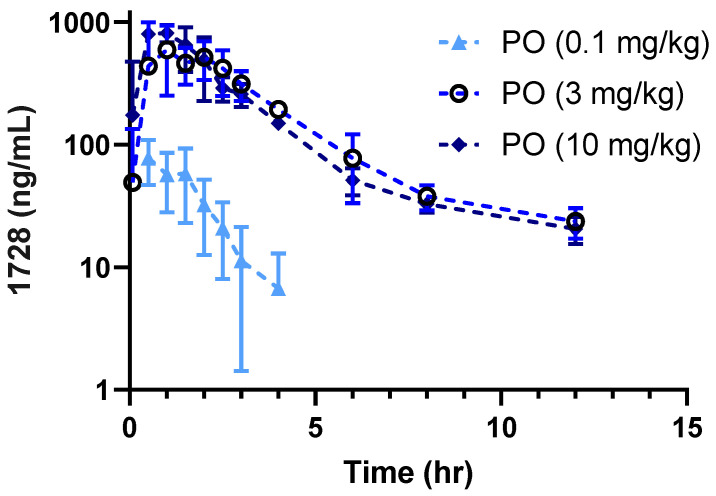
Dose dependent exposure after oral dosing in cats. Fasted cats (*n* = 3) received increasing doses of EC1728. The maximum exposure was achieved at 3 mg/kg, and bioavailability decreased at the high dose without affecting terminal elimination (Table 3).

EC1728 exposure supports once daily oral dosing in mice, dogs, and horses, with a T_1/2_ of >12 h and bioavailability between 42–50% (Table 2). Dogs showed the longest exposure described by a 2-phase absorption with a rapid alpha phase (T_1/2_ 0.09 ± 0.08 h) followed by long beta phase (T_1/2_ 47.00 ± 10 h) (Table 2, Figure 1). Cats showed significantly higher clearance (6× higher than dogs and horses and 3× higher than mice) and lower volume of distribution (Vss) (3.9, 6.7 and 2.9× lower than mice, dogs and horses, respectively) at a low dose of 0.1 mg/kg IV and PO. Cats often display longer exposures to compounds due to fewer Phase I and II metabolizing enzymes, and a low dose was selected initially as a safe dose to characterize PK. Only early timepoints had detectable drug concentrations. The last collection timepoint (T_last_) in cats was 8 h for IV and 12 h for PO; however, the last timepoint with detectable concentration (C_last_) was 3 h for IV and 4 h for PO. For this reason, a higher dose of 1 mg/kg IV and 3 mg/kg PO were administered to better characterize PK. At higher doses (1 mg/kg IV and 3 mg/kg PO), the Cl was lower, although still faster than observed in dogs and horses (3.5 ± 1.1 vs. 0.45 ± 0.38 and 1.2 ± 0.33 mL/min·kg in cats, dogs and horses respectively after accounting for hepatic blood flow rate (Cl (hep)). The Vss was higher at higher doses in cats (2.0 ± 0.8 at 1 mg/kg vs. 0.4 ± 0.2 L/kg at the 0.1 mg/kg dose) and consistent among all species 1.5–2.6 (L/kg for all animals tested). Oral dose exposure studies determined that bioavailability decreased with higher doses (52 ± 23, 29 ± 6 and 8 ± 1 for 0.1, 3 and 10 mg/kg PO dose, respectively) without appreciable changes to the terminal elimination rate 0.64 ± 0.4, 0.28 ± 0.05 and 0.32 ± 0.06 L/h (Figure 2 and Table 3).

Metabolism profiles were investigated to determine why cats display rapid elimination of EC1728. Plasma protein binding (PPB) was evaluated between species to determine if there was higher free drug available for metabolism and elimination in cats versus other species; however, PPB was similar (>96%) among all species tested (Table 2). Phase II metabolism mechanisms are often responsible for differences in elimination and are not usually tested during early screening programs [2]. Even though cats are considered poor at glucuronidation compared to other species due to a lack of two UGT enzymes (UGT1A6 and UGT1A9), Lautz et al. described cats as having a “peculiar expression and activity” of phase II metabolism enzymes [35,36] because they have other UGT and Phase II enzymes that efficiently metabolize xenobiotics. For this reason, stability of EC1728 was assessed in each species with UDPGA, PAPS and GSH, the necessary co-factors for the main phase II metabolism mechanisms. The S9 liver fraction was used for these experiments in order to capture enzymes located in the microsomes and cytosol. These experiments revealed that EC1728 was equally stable in all species tested (98 ± 2.2, 94 ± 4.7, 105 ± 2, 94 ± 1.2% remaining after 1 h incubations in mouse, cat, dog, and horse liver S9 fractions, respectively; Supplementary Table S2). Hepatic clearance accounting for free drug unbound to proteins and hepatic blood flow rates between species further highlighted the differences in clearance between cats (3.5 mL/min/kg) versus dogs (0.57 mL/min/kg) and horses (1.2 mL/min/kg) (Table 2).

Mouse PK was included to correlate multiple published efficacy studies to PK values and relate those values to efficacy in companion animal species. Previously published data evaluated EC1728 after a single oral dose of 0.1, 0.3, 1, and 3 mg/kg and reported dose-dependent increases in AUC and Cmax [37]. An oral dose of 10 mg/kg and IV of 1 mg/kg was selected for novel evaluation in this study and to support previously published studies showing efficacy in a diabetic pain model at increasing subcutaneous doses from 1 to 10 mg/kg, with only 10 mg/kg showing statistical significance [27]. The Cmax and AUC reported here were consistent with previously published data and were only slightly higher than expected (2.6 and 1.3-times higher Cmax and AUC, respectively) based on Liu et al. [37]. Mice also demonstrated 2-phase absorption with a rapid alpha phase (T_1/2_ 1.2 ± 0.34 h) followed by long beta phase (T_1/2_ 43.8 ± 23 h) (Table 2). As expected from rodent species, particularly from mice with exceptionally high heart rates, intrinsic clearance of EC1728 in rodents was more rapid than larger animal species (Table 2).

### 2.2. Predictions of Clearance Based on Allometry

**Figure 3:**
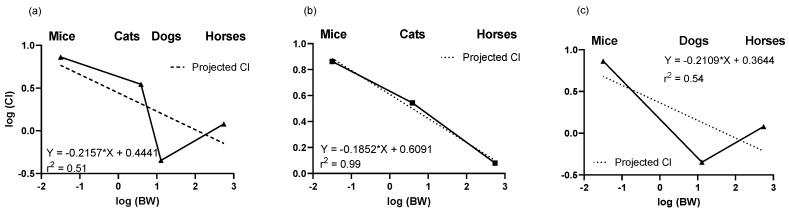
Relationship between clearance vs. body weight. The accuracy of observed clearance was compared to the predicted clearance based on body weight. Clearance was predicted based on the graphing observed clearance values vs. body weight for mice, cats, dogs and horses (**a**), excluding dogs (**b**) or excluding cats (**c**). Excluding dog clearance improved the accuracy of allometric scaling.

Allometric scaling based on body weight was determined for Cl to assess expected vs. observed values. The 1 mg/kg IV dose in cats was used for comparisons to IV hepatic clearance in mice, dogs and horses. Due to the slow Cl in dogs vs. high Cl in cats, there was a high variability in plotting clearance vs. body weight for all species (r^2^ = 0.51) (Figure 3). Accuracy (calculated/predicted) of predicting clearance values based on body weight was calculated from the allometry constant and coefficient determined from all animals or based on calculations where either the cat or dog data was excluded. Based on allometric scaling of mice, dogs, and horses, dog clearance was lower than would be predicted based on body weight (r^2^ = 0.54), whereas clearance in cats compared to mice and horses was as expected based on body weight (r^2^ = 0.99). Although the rapid clearance in cats suggests they are unique in their metabolism, body weight calculations suggests that in fact dogs may be unique in their slow elimination profile.

### 2.3. Efficacy of EC1728 in a Monosodium Urate Model of Inflammatory Pain

**Figure 4:**
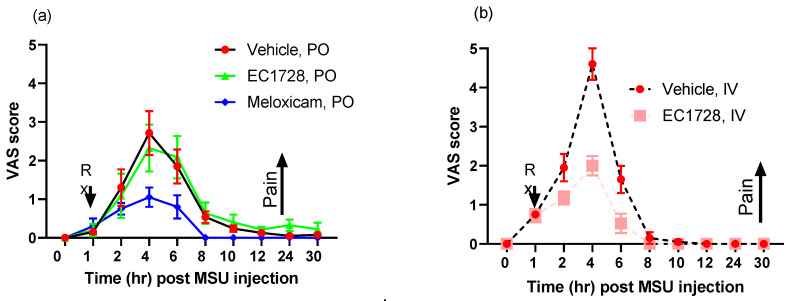

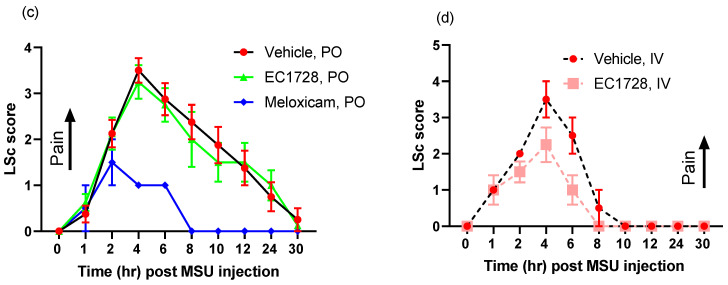
Analgesic activity of EC1728 and meloxicam in cats after MSU injection (mean ± SEM). EC1728 was administered once orally at 3 mg/kg or IV at 0.1 mg/kg 60 min after MSU injection. Meloxicam served as a positive control and was administered once orally at 0.1 mg/kg 60 min after MSU injection. Pain response was recorded by (**a**,**b**) visual analog scale (VAS) or (**c**,**d**) lameness score (LSc). A vehicle control matched each dosing regimen (IV, PO). Oral administration of EC1728 did not alter the pain response after MSU injection, but a single IV dose significantly decreased pain in both recorded pain measures (*p* < 0.05). *n* = 8 for vehicle and EC1728, IV, and *n* = 4 cats were used for EC1728, PO. An *n* = 2 cats were used for IV vehicle and meloxicam since the response was consistent with previous results (not shown) this was deemed sufficient for comparison.

EC1728 is a potent inhibitor of cat sEH with an IC_50_ of 0.4 nM [23], and despite rapid clearance, concentrations in cats still exceeded 10x above the IC_50_ for at least 3-h even at the lowest dose tested. Because of these favorable characteristics, short-term pain models were assessed in spite of the rapid clearance in the PK profile. In a monosodium urate crystal model (MSU) of synovitis, two pain observations were monitored as described in the method section after administration of EC1728. EC1728 at 0.1 mg/kg IV one hour after MSU injection into the stifle joint significantly decreased pain (Figure 4); however, a single oral dose of 3 mg/kg EC1728 after MSU injection failed to show a difference in the pain response (Figure 4). Clinical observations and physical exam found no serious adverse events related to test article administration. Body weights did not deviate from −2 to 7% over the course of the study, and there was no test article related effect on appetite (cats routinely ate 67–100% of food offered). All cats returned to normalcy for all parameters tested 6-days after MSU injection (data not shown).

## 3. Discussion

Understanding PK parameters in target animal species is important for identifying a safe and efficacious dose for treatment. Among the urea based sEH inhibitors potency was initially optimized based on the murine and human recombinant enzyme [38] and then the library rescreened to find potent inhibitors of the canine and equine enzymes [23]. The IC_50′_s of two of the potent sEHIs commonly used in the field compared to EC1728 are shown in Table 1 along with some of their properties. Both compounds are high melting suggesting stable crystal structures as expected of the urea pharmacophore. When the property of high melting point is combined with lipophilicity as indicated by the log *p* values, the materials must be administered in a formulation that will present the inhibitors in true solution because they will dissolve very slowly in biological fluids. Fortunately, as indicated by the low IC_50′_s on the target enzyme, the potency can be very high making the compounds attractive as pharmaceuticals in spite of their challenging physical properties. A surprise in examining the piperidine structures such as EC1770 and AR9281, an sEH inhibitor previously administered in human clinical trials for hypertension [35], was that although these structures were very potent inhibitors of rodent and primate enzymes their potency fell off dramatically in other species, particularly the cat [23]. Thus, when EC1728 was selected as an Investigational New Animal Drug candidate for canine and equine use, we evaluated it for use in felines.

In our assessment of EC1728 exposures in selected animal species, we observed unique PK profiles demonstrating that extrapolations of exposures between species is not the most accurate for this compound. The most notable variability between species was between cats and dogs, with cats displaying faster clearance of EC1728 vs. dogs, and dogs demonstrating much slower clearance than would have been predicted by allometric scaling (Figure 3). Cats are reported to metabolize compounds more slowly than other animals due to having lower levels of CYP450 enzymes, and lack of two glucuronidation (UGT1A6 and UGT1A9) and one ATP-binding cassette transporter (ABCG2) [5,34,35]. While faster metabolism typically represents less risk of toxicity, it makes identifying effective concentrations more challenging. Due to differences in clearance observed in cats and dogs, several obvious elimination pathways were analyzed to understand these differences. Free drug in the plasma can increase elimination due to the availability of the drug to metabolic mechanisms; however, the PPB of EC1728 is consistent across species, and the drug is highly stable in assessments of both Phase I and II elimination mechanisms in vitro. Given the stability of EC1728 after incubation with phase I and II metabolic enzymes from cats with added co-factors, elimination by efflux transporters, or failed binding to absorptive transporters remained as possible explanations as to why clearance rates are more rapid in cats. Additional observations suggest that transporters highly influence cat PK. For example, lower bioavailability in general and at higher oral doses without altering clearance, as observed in a dose escalation study in cats, suggests that transporters, specifically ones with high kcat and poor (high) Km that would be more active at higher concentrations, are responsible for elimination [39]. Additionally, the Biopharmaceutics Drug Disposition Classification System (BDDCS), proposed by Wu and Benet [40] to predict absorption and elimination based on physical properties, identify EC1728 as a Class IV drug with low solubility and low metabolism. Based on this BDDCS classification, EC1728 would be highly absorbed but also influenced by transporters. There is limited information available on specific transporters in cats besides a lack of ATP-binding cassette G2 transporters [5,34,35]; however, based on the stability in vitro in liver microsomes and S9 fractions, a unique species-specific interaction with a transporter may explain the low oral bioavailability in cats and increased exposure in dogs. Due to the use of dogs as the preferred tox species in the development of human drugs, much more is known about the expression of dog transporters; but even in dogs, little is known about the functional role these changes play in the absorption or elimination of xenobiotics [41,42]. It is possible that other factors influenced PK. For example, precipitation of EC1728 in the gut after dosing could contribute to poor bioavailability in cats; however, further studies are needed to confirm this. Future studies comparing the differences in the expression of dog and cat transporters, testing alternate dosing routes that would avoid first pass metabolism in the gut (e.g., topical or IP dosing), or testing different formulations to improve bioavailability would allow for focused investigations on transporter involvement and improvements with formulation.

Despite rapid elimination, EC1728 was effective in reducing pain after a single IV administration, indicating excellent intrinsic potency and validating the target. However, no significant analgesia was seen after oral administration in cats. Efficacy in a short-term pain model is expected considering that EC1728 is a transition state mimic inhibiting the sEH enzyme at low concentrations and with high affinity and high target occupancy due to slow kinetic off-rate from the target enzyme [43]. sEH inhibitors with these characteristics often demonstrate target-mediated drug disposition (TMDD) that results in observed efficacy independent of PK due to elimination largely resulting from the inhibitor binding the intracellular sEH enzyme with high affinity and thus removing it from detection in the plasma while still retaining a high level of enzyme inhibition. Similar sEHI have demonstrated TMDD properties, and these properties are often characteristic of drug classes [44]. Interpretation of efficacy results in compounds demonstrating TMDD are often more complicated than simple correlations of drug exposure translating to efficacious effects and may explain why low exposures of EC1728 after IV dosing results in acute analgesia in an inflammatory pain model in cats. It is harder to explain why oral dosing of EC1728 was not efficacious despite plasma coverage above the IC_50_ during the course of the study. It is possible that Cmax is an important parameter for driving efficacy of EC1728, and the low Cmax after single oral doses was not adequate for driving the compound into target compartments and loading the sEH target with inhibitor. Additional studies investigating target engagement (for example monitoring EpFA: diol ratios in target tissues and not just plasma) would help address this question.

These data are limited in interpretation by the following considerations. Only one breed of dogs was included in this study. Beagle dogs are the most frequently used breed in animal research. Although there are documented PK differences between dog breeds [45], beagles were used to provide comparison to other published literature, and the variability in other breeds would require an unrealistic sample size for this early research. While there are some differences in CYP metabolism between dog breeds [3], due to the metabolic stability of EC1728, future studies would benefit from comparing large versus small breeds to understand PK effects of gut transit times and drug excretion routes for this specific compound. Furthermore, hormonal differences between sexes can alter PK due to hormone effects on both Phase 1 and Phase 2 metabolism enzymes. Both male and female animals were used in these studies; however, considering the metabolic stability of EC1728 demonstrated in the in-vitro experiments, these differences may not be as pronounced as compounds that rely on enzymatic metabolism for clearance. There are also gender differences in pain response; however, the study used equal distribution of female and neutered male animals, and the use of castrated males helps control for these differences [46].

## 4. Materials and Methods

### 4.1. Solubility and Potency

Solubility and potency were determined as previously described [23]. Briefly, solubility was determined at saturation conditions by dissolving 0.25–2 g of compound into PBS (0.1 M, pH 7.4) for 30 °C for 24 h, filtering through a 0.22 uM filter and analyzing the dissolved compounds by LC/MS/MS. Potency was determined using a fluorescent based assay in purified enzymes at a final concentration of 17, 99 and 208 µg/mL for horse, cat and dog, respectively and a final CMPC concentration of 5 µM. Reactions were incubated at 30 °C for 5 min. These conditions were optimized in each species to ensure linear product formation over the course of the experiment. Melting points were determined on an OptiMelt melting point apparatus. 

### 4.2. Test Article and Preparation of Dosing Solution

EC1728 (*t*-TUCB) and internal standard, deuterated 1728 *trans*-4-(4-(3-(4-trifluoromethoxy-phenyl)-ureido)-cyclohexyloxy)-benzoic-2,3,5,6-*d*_4_ acid, also referred to as 3049, *t*-TUCB-*d*_4_), was synthesized as previously described [32,47]. IV dosing solutions were dissolved in DMSO and filtered through a 0.2-micron sterile filter. For mouse (IV) and cat (IV and PO) formulations, PEG400 and saline were added after the compound was dissolved in DMSO and then filtered through a 0.2-micron sterile filter. Oral dosing solutions were dissolved in PEG400 by heating to 50 °C for 10 min followed by 1 min of sonication. Dosing volumes and concentrations are described in Table 4 below.

### 4.3. In Vitro Metabolism and Plasma Protein Binding

**Plasma protein binding:** frozen plasma, pooled mixed sex from mice, cats, dogs, and horses was purchased from Biochemed Services. Plasma protein binding was tested using the Plasma Protein Binding Equilibrium Dialysis (RED device) from Thermo Fisher Scientific (Waltham, MA) following manufacturer’s instructions [29]. Briefly, 100 μL of plasma was spiked with EC1728 (final concentration 1μM in 0.1% DMSO) and loaded in the sample chamber. A negative control containing spiked dialysis buffer was loaded into the sample chamber and used to validate the assay. 350 μL of dialysis buffer (PBS containing 100 mM sodium phosphate and 150 mM sodium chloride) was added to the buffer chamber. Plates were sealed and samples were incubated at 37 °C on an orbital shaker at 250 rpm for 4 h. To terminate the reaction, a sample of the buffer and sample side were removed and added to an equal volume of sample or buffer, respectively. A 6× volume of ice-cold methanol containing internal standard, *t*-TUCB-d4, was added to each tube and centrifuged for 10 min at 13,000× *g* before analysis by LC/MS/MS as described below.

**In-vitro metabolism studies**: Liver S9 fractions, pooled mix gender, were purchased from Sekisui Xenotech and used at a final concentration of 1 mg/mL. Metabolism studies were executed following methods published by Richardson et al. [2]. Briefly, 200 mM Tris buffer containing 2 mM magnesium chloride, pH 7.4 was used for dilution. EC1728 was dissolved in DMSO at 0.2 mM for final concentration of 1 μM. Activating cofactors included phase 1 (NADPH regenerating system) [48] and phase 2 (UDPGA, PAPS, GSH) reactions. S9 fractions and EC1728 were incubated at 5 min before addition of activating co-factors or buffer (control). A separate control without S9 was included to monitor inhibitor stability. Reactions were run in a shaking water bath at 37 °C for 60 min and terminated with an equal volume of ice-cold methanol containing internal standard, t-TUCB-d4, centrifuged for 10 min at 13,000× *g*, and stored at −80 before analysis by liquid chromatography-tandem mass spectrometry (LC/MS/MS) as described below.

### 4.4. In-Vivo Studies

All animal procedures were preapproved by the Institutional Animal Care at each institute conducting the in vivo study (dose administration and blood collection was conducted at University of California at Davis for dogs, horses, mice, and cats administered PK doses at 1 mg/kg, IV. Kingfisher International conducted all other studies in cats reported here). The number and date of receipt of the agreement from bioethical commission of each institution is listed in the Supplementary Materials. Concomitant medications were withheld for a minimum of two weeks prior to administration of EC1728. Dogs, cats and horses were fasted 12-h prior to receiving IV or oral EC1728. Otherwise, animals received *ad libitum* water and routine animal chow throughout the study. Cats, dogs and horses were confirmed healthy by complete blood count, serum biochemistry, and physical exam. Mice were confirmed healthy by visual examination.

Animal dispositions are described below (additional data in Supplementary Table S1)

**Mice**: Four male swiss-webster mice (6–8-week-old from Charles River) with an average ±SD weight of 31 ± 0.1 g were used in each IV and PO arm, for a total of 8-mice.

**Cats**: Three separate studies comprised the cat data. For the PK studies at 0.1 mg/kg and all PO dosing, twelve adult domestic short-hair cats (9 neutered male and 3 intact female) aged 8–33 months with an average ±SD weight of 3.9 ± 0.3 kg were enrolled in this study. To eliminate variability across different experiments, the oral bioavailability was calculated from the 0.1 mg/kg dose in these cats. To better determine terminal clearance, six adult spayed female cats were used for IV dosing at 1 mg/kg with an average ±SD weight of 4.6 ± 0.5 kg. Eight cats were enrolled in efficacy studies with MSU injection (7–35 months, 4 neutered male, 3 female, and 1 spayed female, with an average ±SD weight of 4 ± 0.2 kg).

**Dogs**: Five adult University owned dogs, sexually intact, male 1–2-year-old beagle dogs with an average weight of 12.92 ± 1.6 kg.

**Horses**: Eight University owned horses (four sexually intact females and four castrated males) aged 4–23 years with an average ± SD weight of 555 ± 34.4 kg were used in a cross-over exposure design in this study.

IV injection was administered by tail vein injection in mice and slow injection (30–60 s) via catheter in the cephalic vein of cats and dogs and jugular vein for horses. For oral dosing, mice received liquid oral dosing solution by gavage; cats and dogs received liquid dosing solutions via capsule (Torpac capsule, size 3), and horses by per os by syringe.

Blood samples were collected by venipuncture or pre-placed catheter in the jugular, cephalic or saphenous vein in dogs, cats and horses into EDTA (K_2_) tubes and plasma was isolated by centrifugation and stored at −70 °C until analysis. In mice, blood was collected by tail nick and stored in 0.1% EDTA water frozen at −70 °C until analysis. Blood sampling after IV was collected in a dedicated catheter or separate location from injection to avoid residual compound from injection confounding results. In dogs, blood was sampled from the cephalic vein after oral administration of the test article. Timepoints of blood collection are listed in Table 5. Concentrations of EC1728 were measured by LC-MS/MS as previously described [25,29].

### 4.5. Feline Efficacy Studies

A masked, crossover design was implemented. Eight cats were randomly allocated to two sequence groups to evaluate efficacy after EC1728 IV and oral administration (Table 6). Monosodium urate (MSU) crystals (20 mg in 1 mL suspension) were injected into the stifle joints in cats (left stifle for the first period and right stifle for the second) of anaesthetized cats following the anaesthesia protocol described below. EC1728 was administered by IV injection (period 2) or oral capsule (period 1) 1 h after MSU injection. Meloxicam was included as a positive control and administered at a dose of 0.1 mg/kg 1-h after MSU injection. A vehicle control matched each dosing regimen. Analgesia was assessed by a Visual Analogue Scale (VAS) and Lameness Score (LSc) along with clinical observations, body weight, and physical examination. VAS was scored first and assessed through observation on a scale of 0 (no pain) to 10 (most severe pain). The VAS was designed to assess the degree of overall pain observed for each cat. A mark was made on a continuous (non-graduated) line that had the upper limit labeled as “Severe, pain could not be worse” and the lower limit labeled as “No pain observed”. Marks made on the VAS scoring record were lined up with a transparent scale from 0.0 to 10.0 (with increments of 0.1); thus providing a score between 0.0 and 10.0 for each cat at each observation time point. Scoring on VAS did not involve physical contact with the cat. LSc was rated on a scale of 0 (no observable lameness) to 4 (unable to bear weight). Cats were monitored at 0, 1, 2, 4, 6, 8, 10, and 12 h and 7-, 14-, and 21-days post injection, as well as days 6, 13, and 20 to ensure cats returned to normalcy after MSU injection. Statistics were determined using repeated measure ANOVA mixed effect model using time and dose as variables in Graph Prism version 9.1.

#### Anaesthesia

On the day of MSU injection, the skin over a cephalic vein was clipped as needed, scrubbed with surgical scrub, wiped with isopropyl alcohol, and finally wiped with surgical prep solution. An appropriately sized IV catheter was inserted and taped in place. An injection port was affixed to the catheter and an appropriate amount of 0.9% sterile saline was used to flush the catheter. General anaesthesia was induced and maintained with propofol to effect using the IV catheter and flushed with 0.9% sterile saline. Once the cat was anaesthetized (i.e., no jaw tone or palpebral reflex), lidocaine spray was used on all cats to assist with intubation and an appropriately sized endotracheal tube was placed. Eyes were lubricated and re-lubricated as needed during the procedure. Warming devices were used as needed to help maintain body temperature while under anaesthesia. Anaesthesia was maintained with propofol IV as needed. Anaesthetic procedures and drugs were recorded. 

### 4.6. Pharmacokinetic Parameter Estimation

Individual parameters were calculated by fitting blood concentrations to a non-compartmental analysis using Kinetica software (Thermo Fisher version 5.1, Waltham, MA). Using the log-linear trapezoidal method, the area under the curve were calculated and were extrapolated to infinity using the last measured plasma concentration (Clast), defined as the timepoint collected 72 h after the last dose or 8 h after the first dose on day 1, divided by the terminal slope (λz).

The following PK parameters were estimated, if sufficient data was available:

**AUC_total_**: Area under the plasma drug concentration curve over time. Calculated as mixed log-linear AUCtlast + (Clast/kelim). If the AUCtlast/AUC0-∞ was at least 0.80, the sampling profile was deemed adequate.

**Cmax** and **Tmax**: Highest observed dose (Cmax) at the given time (Tmax). Obtained from experimental observations.

**T_1/2_**: Estimated as the amount of time it takes to eliminate half of the maximal drug concentration. Calculated as ln (2)/kelim.

**k_elim_**: Elimination rate constant. Estimated using linear regression on the terminal phase of the semilogarithmic concentration-time curve. Values below the LLOQ which occur after Tmax were excluded from calculation of the terminal regression line. A minimum of three data points were used for the calculation of kelim.

**F(%):** Absolute oral bioavailability was calculated as ([AUC (PO)_dose (IV)]/[AUC (IV)·dose (PO)])·100.

**Cl(int):** Apparent total clearance of the drug from plasma is calculated as: CL = Dose/AUC_total_

**Cl(hep):** Measure of the maximal clearance in the absence of protein binding or blood flow differences between species is calculated as: Q [(f × Cl(int)/(Q + f × Cl(int)], where Q is the hepatic blood flow rate (mL/min/kg) and f is estimated fraction of EC1728 unbound to plasma proteins.

PK values were evaluated for non-compartmental analysis using the Akaike Information Criterion (AIC) in Graphpad Prism. Multiple compartmental modeling was conducted if the AIC was >0 using a semilog-linear simpler model compared to a segmental regression alternative model.

**Allometric Scaling:** The log value of all pharmacokinetic values was plotted against the log body weight and the linear regression was calculated in Graphpad Prism and used to calculate predicted clearance. The efficiency ratio was determined by the predicted values/observed values [49].

## 5. Conclusions

The PK profile of EC1728 was similar between most species with some important differences: intravenous and oral administration revealed that bioavailability was similar for dogs, and horses (42 and 50%F) but lower in mice and cats (34 and 8%, respectively). Additionally, clearance was similar between cats and mice, but >2× faster in cats vs. dogs and horses. Yet despite the faster clearance, EC1728 demonstrated efficacy in an acute pain model in cats after IV but not PO administration. These results demonstrate that exposures across species can vary, and investigation of therapeutic exposures in target species is needed to provide adequate care that addresses efficacy and avoids toxicity.

In both human and animal drug development, translation of efficacious concentrations is often based upon rodent PK data or correlations from other species. Therefore, the data in this publication further emphasizes that PK cannot be assumed based on general assumptions of body weight and hepatic blood flow rates. While an understanding of companion animal PK can advance safety and efficacy for companion animal drugs, it can also advance an understanding of fundamental PK parameters since unique differences in animal species aid to better characterize chemical classes as they relate to metabolism, and intrinsic characteristics that affect distribution and elimination. Although EC1728 is in development as an oral analgesic for dogs, for inflammatory pain for equine arthritis, and IV analgesic for equine laminitis (neuropathic pain), its use at this point for cats is likely to be limited to IV administration unless a unique formulation can be found to increase prolonged blood levels of the drug. It is possible that an understanding of the low blood levels and the mechanism for rapid elimination of the drug in cats will provide a pathway. Ultimately, the rapid clearance of EC1728 provides a unique opportunity to investigate differences in PK between species and, understand important PK parameters required for efficacy, or unique handling challenges that need to be taken into consideration when working with feline pain models.

## Figures and Tables

**Table 1:** Summary of solubility and potency of sEH compounds.

Compound	Solubility (µg/mL) ^a^	Melting Point (°C)	cLogP ^b^	Potency (Ki) (ng/mL) ^c^			
				Mouse	Cat	Dog	Horse
EC1728 (*t*-TUCB) 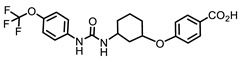	5	240–244 (242)	4.7	11	0.18 ± 0.004	0.39 ± 0.02	25.87
**Piperidine Series**							
UC1770 (TPPU) 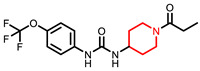	60	198.2–200.8 (199.5)	1.5	2.9 ± 0.72	155 ± 80	1138 ± 696	25 ± 9
AR9281 (UC1153, APAU) 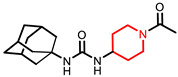	277	205–206 (205.5)	0.8	2.8	144	160	39

^a^ The solubility of the drugs was measured in 0.1 M Sodium Phosphate Buffer at pH 7.4. ^b^ cLogP was calculated using ChemBioDraw (v 19.1 CambridgeSoft). ^c^ IC_50_ was calculated using CMNPC at 5 µM as previously described [32].

**Table 2:** Summary of key PK parameters between species.

Parameter ^1^	Mouse	Cat		Dog	Horse
**Dose (mg/kg)**					
IV	1	0.1	1	0.3	1
PO	10	0.1	3	0.3	1
**Cmax (ng/mL)**					
IV	3087 ± 1089	2076 ± 1194	27,241 ± 8114	1566 ± 1128	2436 ± 304
PO	2570 ± 670	91.5 ± 14.6	802 ± 241	403 ± 232	336 ± 83
**IV-Dose adjusted** (Cmax/dose)	3087 ± 1089	20,760 ± 11,942	27,241 ± 8114	5219 ± 3761	2436 ± 304
**PO-Dose adjusted** (Cmax/dose)	257 ± 67	915 ± 146	267 ± 80	1343 ± 772	336 ± 83
**AUC (h·ng/mL)**					
IV	8488 ± 1319	271 ± 88	8182 ± 3083	12,662 ± 8309	16,604 ± 4535
PO	28,624 ± 6008	140 ± 62	2081 ± 137	14,443 ± 4852	9190 ± 2921
**IV-Dose adjusted** (AUC/dose)	8488 ± 1319	2716 ± 878	8182 ± 3083	96,388 ± 63,239	16,604 ± 4535
**PO-Dose adjusted** (AUC/dose)	2862 ± 601	1402 ± 619	693 ± 46	48,142 ± 16,173	9190 ± 2921
**Tmax (h)**					
IV	0.38 ± 0.14	0.04 ± 0.04	0.025 ± 0	0.04 ± 0.03	0.08 ± 0
PO	4.0 ± 0.0	0.8 ± 0.6	1.2 ± 0.8	10.3 ± 9.3	10.6 ± 3.8
**Cl_int_ (mL/min·kg)**	2.0 ± 0.3	6.6 ± 2.1	2.3 ± 0.8	0.7 ± 0.6	1.0 ± 0.3
**Vss (L/kg)**	1.5 ± 0.9	0.4 ± 0.2	2.0 ± 0.8	2.6 ± 2.0	1.2 ± 0.2
**%F**	34 ± 8	52 ± 51	29 ± 6 *	42 ± 14	50 ± 8
**T_1/2_ (h)**		1.4 ± 0.4			16.5 ± 2.3
**IV α**	α = 1.20 ± 0.34		α = 0.23 ± 0.13	α = 0.10 ± 0.08	
**β**	β = 44 ± 23		β = 20.5 ± 7.4	β = 47 ± 10	
**PO**	16.3 ± 2.8	0.9 ± 0.2	4.6 ± 4.1	42 ± 20	18.0 ± 4.3
**PPB (%)**	96.04 ± 0.99	98.32 ± 0.61	98.32 ± 0.61	98.0 ± 1.4	98.75 ± 0.24
**Cl(hep) (ml/min·kg)**	7.30 ± 0.97	8.60 ± 2.12	3.5 ± 1.1	0.45 ± 0.38	1.2 ± 0.33
**Body weight (kg)**	0.032 ± 0.002	3.9 ± 0.3		12.9 ± 1.6	554 ± 22

Numbers represent mean ± STDEV. ^1^ PPB (Plasma Protein Binding), IV (intravenous), PO (oral gavage in mice and per os for cats, dogs and horses), Cl_int_ (intrinsic clearance), %F (oral bioavailability), T_1/2_ (half-life), Cl (hep) (hepatic clearance). Hepatic blood flow rates used to calculate Cl (hep) are 30, 40, 35, 24 mL/min in mouse, cat, dog and horse, respectively [33,34]. Gender and animals dosed/group are described in the methods section. %F for 3 mg/kg PO in cats was calculated based on the 0.1 mg/kg IV dose group.

**Table 3:** Summary of key PK parameters after oral dosing in cats.

Route	PO		
Parameter ^1^			
**Dose (mg/kg)**	0.1	3.0	10.0
**Cmax (ng/mL)**	92 ± 15	803 ± 241	1115 ± 541
**AUC (h·ng/mL)**	140 ± 62	2369 ± 517	2278 ± 245
**%F Based on 0.1 mg/kg**	52 ± 23.0	29 ± 6.0	8 ± 1.0
**T_1/2_ (h)**	0.88 ± 0.2	4.62 ± 4.1	4.50 ± 2.2
**Tmax (h)**	0.83 ± 0.6	1.17 ± 0.8	0.83 ± 0.3
**Cl_int_ (mL/min·kg)**	14.4 ± 8.4	24.1 ± 1.6	73.7 ± 7.8
**Kel (1/h)**	0.64 ± 0.35	0.28 ± 0.05	0.32 ± 0.06

^1^ Numbers represent mean ± STDEV. IV (intravenous), PO (per os), Cl_int_ (intrinsic clearance), %F (oral bioavailability), T_1/2_ (half-life).

**Table 4:** Concentration and dosing volumes of dosing solutions.

	Vehicle	Dose (mg/kg): Concentration (mg/mL)
**Mouse**	IV: DMSO:PEG300:Saline (2:25:73) PO: PEG400 (100%)	1.0 mg/kg: 0.06 mg/mL, IV 10 mg/kg: 1 mg/mL, PO
**Cat**	IV (0.1 mg/kg) and PO: DMSO: PEG400 (20:80) PEG400 for 1 mg/kg IV	0.1 mg/kg: 2 mg/mL, IV and PO 1 mg/kg: 20 mg/mL, IV 3.0 mg/kg: 60 mg/mL, PO 10 mg/kg: 100 mg/mL, PO
**Dog**	IV: PEG400 (100%) PO: PEG400 (100%)	0.3 mg/kg: 1 mg/mL, IV 0.3 mg/kg: 1 mg/mL, PO
**Horse**	IV: DMSO (100%) PO: PEG400 (100%)	1 mg/kg: 20 mg/mL, IV 1 mg/kg: 20 mg/mL, PO

**Table 5:** Blood samples times post dose.

Species	Time (h)
Mouse	IV: 0.25, 0.5, 1, 2, 4, 6, 8, 12, 24, 48, 72, 96 (*n* = 4) PO: 0.25, 0.5, 1, 2, 4, 6, 8, 12, 24, 48, 72, 96 (*n* = 4)
Cat	IV (0.1 mg/kg): 0.017, 0.08, 0.25, 0.5, 0.75, 1, 1.5, 2, 3, 6, 8 (*n* = 3) IV (1 mg/kg): 0.025, 0.05, 0.1, 0.2, 0.38, 0.75, 1.5, 3, 6, 12, 24, 36, 48, 72, 96 (*n* = 6) PO: 0.08, 0.5, 1, 1.5, 2, 2.5, 3, 4, 6, 8, 12 (*n* = 3 per group)
Dog	IV: 0.025, 0.05, 0.1, 0.2, 0.38, 0.75, 1.5, 3, 6, 12, 24, 36, 48, 72, 96 (*n* = 5) PO: 0.025, 0.05, 0.1, 0.2, 0.38, 0.75, 1.5, 3, 6, 12, 24, 36, 48, 72, 96 (*n* = 5)
Horse	IV: 0.083, 0.167, 0.25, 0.5, 0.75, 1, 2, 3, 4, 6, 8, 12, 18, 24, 30, 36, 48, 60, 72, 96 (*n* = 8) PO: 0.083, 0.167, 0.25, 0.5, 0.75, 1, 2, 3, 4, 6, 8, 12, 18, 24, 30, 36, 48, 60, 72, 96 (*n* = 8)

**Table 6:** Efficacy study design in cats administered monosodium urate and treated with EC1728.

Sequence Group	Period 1	Wash-Out	Period 2
S1 (*n* = 4)	Vehicle ^€^	7 days	Test Article ^£^
S2 (*n* = 4)	Test Article *		Reference Article ^α^ or Vehicle ^β^

* One oral dose of EC1728 at 3.0 mg/kg (between 50 and 60 min post MSU injection); € Equivalent frequency, timing, and volume to the respective test article counterpart; £ One IV dose of EC1728 at 0.1 mg/kg (between 50 and 60 min post MSU injection); α Cohort 1 (*n* = 2) received one oral dose of METACAM at 0.1 mg/kg as a positive control (between 50 and 60 min post MSU injection); β Cohort 2 (*n* = 2) received one IV dose of vehicle (between 50 and 60 min post MSU injection).

## Data Availability

Not applicable.

## Supplementary Materials

The following are available online, Supplementary data contains the following information: Individual animal PK parameters (Table S1), semi log-linear PK representations through 12-h (Figure S1), stability in S9 liver fractions (Table S2), allometric scaling predictions in all species tested (Table S3).

## Sample Availability

Samples of the compounds EC1728 are available from the authors.
