# Supplementary material for: Species Differences in Metabolism of Soluble Epoxide Hydrolase Inhibitor, EC1728, Highlight the Importance of Clinically Relevant Screening Mechanisms in Drug Development
Source: Molecules. 2021 Aug 19;26(16):5034. doi: 10.3390/molecules26165034 (PMC8399023; doi:10.3390/molecules26165034)
Supplement: Supplementary file 1 [file molecules-26-05034-s001.zip › molecules-1326300-supplementary.pdf]

Supplementary materials

Species differences in metabolism of soluble epoxide hydrolase inhibitor, EC1728, highlight the importance of clinically relevant screening mechanisms in drug development.

Cindy McReynolds[1,2], Jung Yang[1,2], Alonso Guedes[3], Christophe Morisseau[1], Roberto Garcia[4], Heather Knych[5,6], Caitlin Tearney[3], Briana Hamamoto[5], Sung Hee Hwang[1,2], Karen Wagner[1,2], Bruce Hammock[1,2]*

[1]Department of Entomology and Nematology, UC Davis Comprehensive Cancer Center, University of California, Davis, Davis, CA, United States,

[2]EicOsis, Davis, CA, United States

[3]Department of Veterinary Clinical Sciences, College of Veterinary Medicine, University of Minnesota, St. Paul, MN 55108, USA.

[4]Dechra Development LLC, 1 Monument Sq, Portland, ME 04101.

[5]K.L. Maddy Equine Analytical Pharmacology Laboratory, School of Veterinary Medicine, University of California, Davis, USA.

[6]Department of Veterinary Molecular Biosciences, School of Veterinary Medicine, University of California, Davis, USA.

*Correspondence: bdhammock@ucdavis.edu; Tel.: (530) 752-8465

Supplementary data includes calculated PK values (Cmax, Tmax, Tlas, AUC_{tot}, Lz, $T_{1/2}$, MRT, Clearance and V_{SS}) for each animal, relationship and projections between clearance, volume of distribution, and $T_{1/2}$ vs. body weight.

Supplementary Table S1. Individual PK parameters

Mouse

IV	Dose	Cmax	Tmax	TLast	AUCtot	Kel	$T_{1/2}$	MRT	Clearance	Vss
Unit	mg/kg	ng/mL	h	h	ng/mL*h	1/h	h	h	mL/min/kg	L/kg
Animal										
1.00	1	3401	0.50	48	8615	0.35	1.96	7.02	1.93	0.81
2.00	1	3988	0.25	96	10305	0.26	2.70	12.65	1.62	1.23
3.00	1	3456	0.25	48	7598	0.17	4.05	9.57	2.19	1.26
4.00	1	1503	0.50	72	7433	0.24	2.93	21.01	2.24	2.83

PO	Dose	Cmax	Tmax	TLast	AUCtot	Kel	$T_{1/2}$	MRT	F
Unit	mg/kg	ng/mL	h	h	ng/mL*h	1/h	h	h	%
Animal									
1.00	10	1802	4.00	96	36251	0.04	15.78	22.38	42
2.00	10	3431	4.00	96	24129	0.05	13.42	20.55	23
3.00	10	2594	4.00	96	30596	0.03	20.17	26.39	40
4.00	10	2453	4.00	96	23523	0.04	15.87	16.65	32

Cat

IV	Dose	Cmax	Tmax	TLast	AUCtot	Kel	$T_{1/2}$ alpha	$T_{1/2}$ beta	MRT	Clearance	Vss
Unit	mg/kg	ng/mL	h	h	ng/mL*h	1/h	h	h	h	mL/min/kg	L/kg
JAJ31[nm]	0.1	708	0.080	3	192.53	0.59	1.18	n.d.	1.05	8.66	0.55
JEC2[nm]	0.1	2910	0.017	8	366.11	0.38	1.82	n.d.	1.41	4.55	0.38
LAU4[nm]	0.1	2610	0.017	3	256.02	0.57	1.22	n.d.	0.66	6.51	0.26
1[f]	1.0	37,267	0.025	96	10131.1	0.02	0.21	21.22	11.2	1.65	1.77
2[f]	1.0	29,028	0.025	96	13344.8	0.03	0.35	19.62	12.8	1.25	1.20
3[f]	1.0	31,399	0.025	96	6820.16	0.04	0.12	12.69	9.0	2.44	1.61
4[f]	1.0	29,751	0.025	96	7355.12	0.03	0.44	34.10	8.0	2.27	1.69
5[f]	1.0	21,948	0.025	96	6817.12	0.03	0.12	15.32	11.3	2.44	1.93
6[f]	1.0	14,055	0.025	96	4622.26	0.03	0.13	19.85	12.6	3.61	3.60

PO	Dose	Cmax	Tmax	TLast	AUCtot	Kel	$T_{1/2}$	MRT	F
Unit	mg/kg	ng/mL	h	h	ng/mL*h	1/h	h	h	%
EAB2[nm]	0.1	80.3	0.5	2.5	69.26	0.92	0.67	1.19	8%
EAQ4[f]	0.1	86.2	1.5	4	168.00	0.75	0.92	1.849	21%
LBB3[nm]	0.1	108	0.5	4	183.27	0.25	1.07	1.859	22%
CBD2[f]	3	642	1.0	12	1944.23	0.32	2.16	3.41	8%
LBA3[nm]	3	1080	0.5	12	2217.88	0.30	2.30	2.52	9%
LBB2[nm]	3	686	2.0	12	2083.26	0.22	3.13	4.47	8%
CBD3[f]	10	1720	0.5	12	2535.76	0.28	2.48	2.48	3%
JEH2[nm]	10	677	1	12	2048.27	0.29	6.91	5.12	3%
LAQ1[nm]	10	948	1	12	2250.28	0.38	4.11	3.10	3%

n.d. = not determined; nm = neutered male; sf = spayed female; f = female; nm = neutered male

Dog

IV Unit	Dose mg/kg	Cmax ng/mL	Tmax h	TLast h	AUCtot ng/mL*h	$T_{1/2}$ alpha h	$T_{1/2}$ beta h	MRT h	Clearance mL/min/kg	Vss L/kg
1	0.3	2358	0.025	96	15268	0.05	55.13	38.95	0.33	1.61
2	0.3	685	0.1	96	14183	0.25	48.47	35.10	0.35	1.27
3	0.3	1024	0.025	96	6337	0.07	41.03	36.56	0.79	3.06
4	0.3	617	0.025	96	3164	0.05	33.55	33.08	1.58	6.00
5	0.3	3145	0.025	96	24359	0.05	58.04	38.84	0.21	1.30

PO Unit	Dose mg	Cmax ng/mL	Tmax h	TLast h	AUCtot ng/mL*h	Kel 1/h	thalf h	MRT h	F %
1	0.3	197	12.00	96	11,825	0.02	33.05	54.69	34
2	0.3	387	24.00	96	12,969	0.02	31.74	46.69	40
3	0.3	157	12.00	96	8,877	0.01	49.82	70.51	61
4	0.3	621	3.00	96	21,212	0.03	21.97	29.80	*294**
5	0.3	656	0.38	96	17,330	0.01	72.89	101.87	31

* Not included in %F average.

Horse

IV Unit	Dose mg/kg	Cmax ng/mL	Tmax h	TLast h	AUCtot ng/mL*h	Kel 1/h	thalf h	MRT h	Clearance mL/min/kg	Vss L/kg
**Animal**										
Flora	1	2506	0.08	96	22249	0.04	16.10	22.34	0.75	1.00
Kitty	1	2284	0.08	72	16242	0.06	11.02	18.02	1.03	1.11
Rice	1	2151	0.08	96	17120	0.04	15.44	20.89	0.97	1.22
Curtis	1	2182	0.08	96	19598	0.04	18.81	22.83	0.85	1.17
George	1	2812	0.08	96	14188	0.04	17.77	15.92	1.17	1.12
Que	1	2910	0.08	96	24562	0.04	18.81	22.66	0.68	0.92
Robin	1	2304	0.08	96	14106	0.04	17.38	19.57	1.18	1.39
Ketchup	1	2406	0.08	72	10388	0.06	12.47	14.27	1.60	1.37

PO Unit	Dose mg/kg	Cmax ng/mL	Tmax h	TLast h	AUCtot ng/mL*h	Kel 1/h	thalf h	MRT h	F %
**Animal**									
Flora	1	311	8.00	96	10176	0.03	21.96	33.90	
Kitty	1	341	8.00	96	7637	0.04	18.50	27.50	55.13
Rice	1	459	8.00	96	9585	0.04	19.70	28.04	48.47
Curtis	1	244	12.00	96	7480	0.06	11.09	26.90	41.03
George	1	262	18.00	96	6884	0.04	16.07	31.15	33.55
Que	1	437	12.00	96	15198	0.05	14.94	29.77	58.04
Robin	1	295	8.00	96	7372	0.03	23.91	36.96	
Ketchup		Not detectable							

Supplementary Figure S1

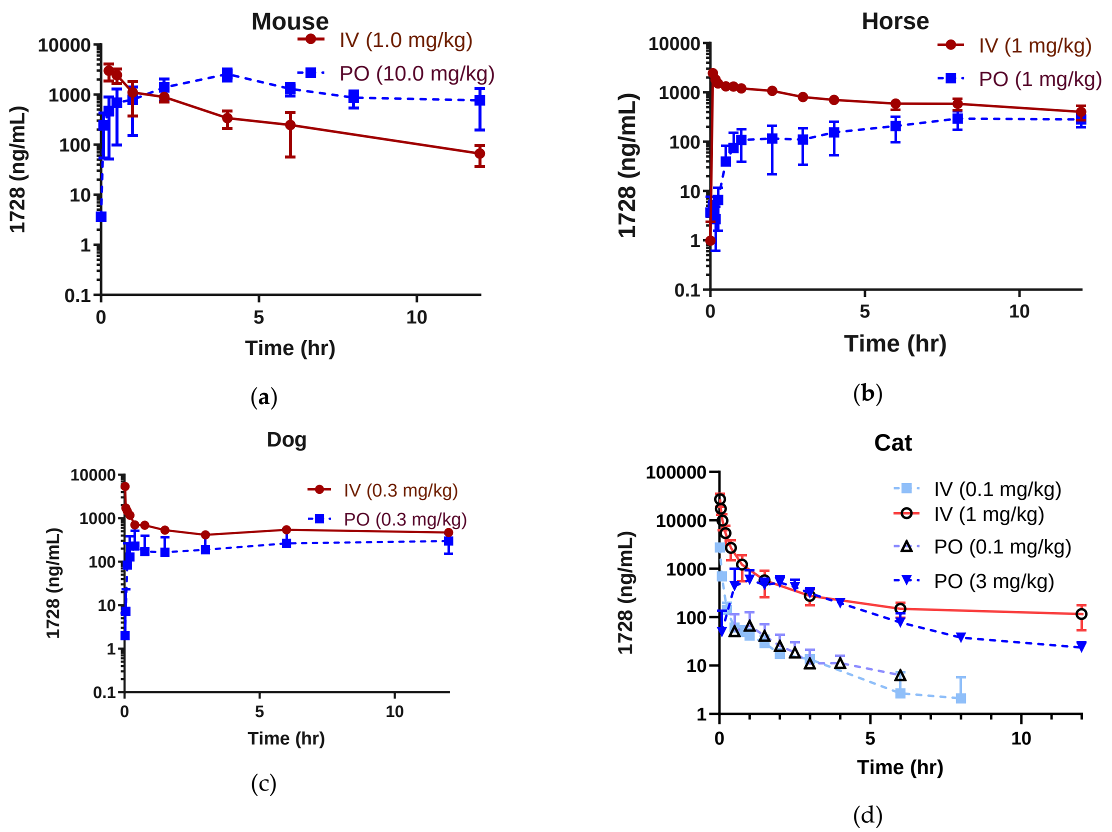

Supplementary Figure 1. Main figure 1 regraphed to emphasize the first 12 hours of distribution and elimination. General overview of PK profiles of EC1728 represented as semi log-linear concentrations after dosing IV or PO in mice (a), horses (b), dogs (c) and cats (d). T_{last} in cats at 0.1 mg/kg iv and PO was 8 hour for IV and 12 hour for PO; however, the C_{last} in the low dose cat group dosed IV was observed at 3 hour and 4 hour for PO.

Supplementary Table S2. Stability of EC1728 in liver s9 fractions isolated from different species

	S9+NADPH+Cofactors (Phase 2 metabolism)	S9+NADPH (Phase 1 metabolism)	S9 (blank)
**Mouse**	25.08	24.56	25.95
	25.86	24.52	25.45
	24.83	24.93	
**% remaining**	98 ± 2%	95 ± 0.1	100 ± 1
**Cat**	23.99	25.08	27.03
	25.90	25.61	26.77
	26.07	27.02	
**% remaining**	94 ± 5	94 ± 1	100 ± 0.1
**Dog**	25.17	25.34	24.28
	26.14	25.87	24.66
	25.46	22.57	
**% remaining**	105 ± 2	107 ± 4	100 ± 1
**Horse**	22.76	22.86	22.62
	23.10	24.13	26.30
	23.34	24.56	
**% remaining**	94 ± 1	95 ± 3	100 ± 11

Numbers represent the peak area of EC1728 divided by the internal standard (deuterated-EC1728). Negative control samples were included without microsomes (25.12, 24.165, 24.49, or 101% remaining). Percent remaining was calculated by dividing average values by average values of s9 sample without cofactors (NADPH, UDPGA, PAPS, GSH).

Supplementary Table S3. Calculated clearance and accuracy based on body weight

	**Predicted Values**		
Species for calculation*	Cl(hep) (ml/min*kg)		
	MCDH	MCH	MDH
Mouse	5.84	7.69	4.78
Cat	2.07	3.16	1.74
Dog	1.60	2.53	1.35
Horse	0.71	1.26	0.61
	**Accuracy**		
Mouse	125%	95%	153%
Cat	169%	111%	202%
Dog	28%	18%	33%
Horse	169%	95%	197%

*M: Mouse, C: Cat, D: Dog, H: Horse

The number and date of receipt of the agreement from bioethical commission of each institution is listed below.

Horses
The project identification code: #20319
Date of approval: 11 November 2018
Ethics Committee: UC Davis Institutional Animal Care and Use committee

Mice
The project identification code: #19296
Date of approval: 3 May 2018, renewed annually.
Ethics Committee: UC Davis Institutional Animal Care and Use committee

Cats:
UC Davis
The project identification code: 17211
Date of approval: 15 November 2012
Ethics committee: UC Davis Institutional Animal Care and Use committee

Kingfisher International
Project Identification Number: KFI-056-KF-3517, date of approval 22 Sep 2017 and KFI-056-EF-0118, date of approval 11 May 2018
Ethics committee: Kingfisher International Animal Care and Use Committee

Dogs:
The project identification code: 17765
Date of approval: 16 September 2013
Ethics committee: UC Davis Institutional Animal Care and Use committee
